# National Eye Institute’s (NEI) coordination efforts and current opportunities for sustainability, adaptation, and climate resilience in global eye health – ARVO 2023 session commentary

**DOI:** 10.1038/s41433-023-02854-9

**Published:** 2023-11-30

**Authors:** Eleanor McCance, Hugh R. Taylor, Nisha R. Acharya, Cassandra L. Thiel, Serge Resnikoff, Rupert Bourne

**Affiliations:** 1 Department of Ophthalmology, Royal Glamorgan Hospital, Llantrisant, UK; 2 Melbourne School of Population and Global Health, Melbourne, VIC Australia; 3 Departments of Ophthalmology and Epidemiology, University of California, San Francisco, USA; 4 Department of Population Health & Ophthalmology, New York University, New York, NY USA; 5 School of Optometry and Vision Sciences, University of New South Wales, Sydney, NSW, Australia; 6 Cambridge Eye Research Centre, Addenbrookes Hospital, Cambridge, UK

**Keywords:** Conferences and meetings, Epidemiology

Climate change represents an urgent and severe threat to global health [1], directly and indirectly driving detrimental physical and mental health. Climate change impacts all countries, but disproportionately affects low- and middle- income countries, exacerbating worldwide health inequalities.

Rising temperatures, sea levels and carbon dioxide levels lead to increasingly frequent extreme weather events (heatwaves, floods, and storms), air pollution, disruption of food and water systems, disruption to healthcare delivery systems and medical supply chains, increased zoonoses, food-, water-, and vector-borne diseases affecting multiple organ systems, including the eyes. Climate-changed induced forced migration is likely to disrupt and overwhelm healthcare infrastructure and supply chains, making it more difficult to achieve universal eye health coverage [2].

In 2019, WHO identified that at least 2.2 billion people worldwide have a vision impairment, and of these, at least 1.1 billion people have a vision impairment that could have been prevented or is yet to be addressed [3]. The leading causes of moderate and severe vision impairment (MSVI) globally are uncorrected refractive error (157.49 million), followed by cataract (83.48 million) [4]. Despite the rate of cataract surgery increasing and the age-standardised prevalence of MSVI and blindness associated with cataract reducing as a result, the number of people with vision loss associated with cataract has increased, due to changing age structure of populations, especially among women [5]. The issues and consequences are more profound in the developing world in comparison to the developed world, with pre-existent inequalities in health-care access, resources and delivery exacerbated by the effects of climate change.

Emerging evidence suggests that air pollution, such as wildfire smoke (particulate matter 2.5 and 10), is not only linked to exacerbation of ocular surface diseases and severe allergic eye disease, but also cataract formation [6, 7], increased risk of developing primary open angle glaucoma [8], diabetic retinopathy [9], and age-related macular degeneration [10]; all conditions that contribute significantly to the burden of vision impairment worldwide. Rising temperatures also exacerbate inflammatory eye disease, and susceptibility to bacterial and fungal infections, and triggers reactivation of viral conditions which can affect ocular tissues [11]. Climate change is altering traditional boundaries for infectious diseases, increasing the susceptible geographical reach, and incidence of infectious diseases manifesting in the eye transmitted by ticks and mosquitoes, such as zika, yellow fever, Lyme, and dengue [12]. Climate change is derailing previous progress in the fight against avoidable global blindness from trachoma, onchocerciasis and vitamin A deficiency, with higher temperatures linked to increased trachoma infection [13], disruption of infrastructures and food supply chains (agriculture affected directly from temperature/ rainfall changes, and indirectly from mismanaged human systems), leading to malnutrition and blindness from xerophthalmia [11]. Climate change-induced migration with resultant environmental degradation, over-crowding, resource depletion and reduced sanitation will add fuel to the fire.

The healthcare sector is responsible for 5% of global greenhouse gas emissions (GHGs), and is a major source of waste and pollution globally, producing 2 gigatons of carbon dioxide equivalent (CO2e). Cataract surgery is the most performed surgical procedure worldwide, and the carbon footprints vary regionally based on the local surgical practice. In the UK for example, the carbon footprint of one cataract operation is estimated to be 181.8 kgCO_2_e, with over 50% of GHG emissions from the procurement of largely single-use disposables [14]. The Aravind Eye Care System in India by comparison estimates that 6 kg/CO_2_ is generated per case (just 5% of the UK’s Phaco ‘carbon footprint’), with comparable complication and endophthalmitis rates [15].

Coordinated global action is required to achieve net zero carbon healthcare (60 countries to date from COP26 have committed to this goal) and increase the sector’s climate resilience. Multilevel global adaptation, mitigation of human activity, and implementation of sustainable practice, including a rethink and rationalise, refuse, reduce, reuse, repair and recycle framework, will be important in achieving the 17 interdependent Sustainable Development Goals (SDGs) set out by the United Nations (UN). The World Health Organisation (WHO) world report on vision proposed an integrated people-centred eye care framework for a structured approach to meet the world population’s needs and meet sustainable development goals through four main pillars; (i) engaging and empowering people and communities; (ii) reorienting the model of care; (iii) coordinating services within and across sectors; and (iv) creating an enabling environment, specifically the inclusion of eye care in national health strategic plans.

Online resources, such as ‘EyeSustain.org’, and the ‘International Agency for the Prevention of Blindness Climate Action Working Group’ (IAPB CAWG) aligns with the UN, WHO, and Lancet Commission on Global Eye Health’s mobilisation of workers in the healthcare sector, engaging the eye health community to work towards sustainable development goals. These resources also help guide climate impact mitigation, so we might meet the demand within our services, and not at the demise of our planet. Integration of environmental strategies will not only promote better health through mitigating the catastrophic effects of climate change, but it will also increase service quality, productivity, and reduce costs, with indirect co-benefits on the physical and mental health of all service-users.

We are at a critical moment in determining the future of our planet. This 2023 ARVO special session organised by NEI, National Institutes of Health (NIH) on climate change highlighted the existing knowledge in the area and an immediate need to appreciate and understand how detrimental it can be to eye health. The healthcare sector is a major contributor to the climate crisis, thus has an important role to play in tackling it and must be mobilised to do so. Inaction risks calamitous geopolitical and financial effects from mass migration, including conflicts and resource depletion which in turn threatens global and eye health. NEI is encouraging global coordination in researching the effects of climate change on eye diseases and on the infrastructures needed to support eye health for patient populations, especially climate refugees. The NIH climate change and health initiative provides an insight into these global coordination efforts.

This session has inspired confidence that workers in the eye care and research sectors are aware of the potential consequences of climate change and are willing to act on mitigating our climate impact and adapting to a changing climate, joining the UNSDG’s commitment to ‘leave no one behind’. What would be left if we do not?
